# Ethical, Legal and Social Issues (ELSI) Associated with Non-Invasive Prenatal Testing: Reflections on the Evolution of Prenatal Diagnosis and Procreative Choices

**DOI:** 10.3390/genes12020204

**Published:** 2021-01-30

**Authors:** Simona Zaami, Alfredo Orrico, Fabrizio Signore, Anna Franca Cavaliere, Marta Mazzi, Enrico Marinelli

**Affiliations:** 1 Department of Anatomical, Histological, Forensic and Orthopedic Sciences, Sapienza University of Rome, 00161 Rome, Italy; simona.zaami@uniroma1.it (S.Z.); enrico.marinelli@uniroma1.it (E.M.); 2 Molecular Diagnosis and Characterization of Pathogenic Mechanisms of Rare Genetic Diseases, Azienda Ospedaliera Universitaria Senese, 53100 Siena, Italy; 3 Clinical Genetics, ASL Toscana SudEst, Ospedale della Misericordia, 58100 Grosseto, Italy; 4 Department of Obstetrics and Gynaecology, S. Eugenio Hospital, 00144 Rome, Italy; fabrizio.signore@aslroma2.it; 5 Department of Obstetrics and Gynecology, Santo Stefano Hospital, Prato USL Toscana Centro, 59100 Prato, Italy; afcavaliere@hotmail.com; 6 Physiopathology of Reproduction Unit, USL Toscana SudEst, Ospedale della Misericordia, 58100 Grosseto, Italy; marta.mazzi@uslsudest.toscana.it

**Keywords:** non-invasive prenatal testing, cell-free fetal DNA, screening, aneuploidies, ethical, legal and social implications

## Abstract

New technologies such as non-invasive prenatal testing (NIPT), capable of analyzing cell-free fetal DNA in the maternal bloodstream, have become increasingly widespread and available, which has in turn led to ethical and policy challenges that need addressing. NIPT is not yet a diagnostic tool, but can still provide information about fetal genetic characteristics (including sex) very early in pregnancy, and there is no denying that it offers valuable opportunities for pregnant women, particularly those at high risk of having a child with severe genetic disorders or seeking an alternative to invasive prenatal testing. Nonetheless, the ethical, legal and social implications (ELSI) include multiple aspects of informed decision-making, which can entail risks for the individual right to procreative autonomy, in addition to the potential threats posed by sex-selective termination of pregnancy (in light of the information about fetal sex within the first trimester), and the stigmatization and discrimination of disabled individuals. After taking such daunting challenges into account and addressing NIPT-related medicolegal complexities, the review’s authors highlight the need for an ethically and legally sustainable framework for the implementation of NIPT, which seems poised to become a diagnostic tool, as its scope is likely to broaden in the near future.

## 1. Introduction

Non-invasive prenatal testing (NIPT), also referred to as cell-free DNA (cfDNA) testing and non-invasive prenatal screening (NIPS), is a highly sensitive and specific screening technique, increasingly clinically adopted to assess the risk that the fetus may carry chromosome aneuploidies and, possibly, submicroscopic copy number variations (CNVs) and other genetic conditions [1]. NIPT takes into account fetal DNA fragments resulted from placental trophoblast apoptosis, i.e., programmed cell death [2], flowing through the maternal blood and detectable from the fifth week of gestation. 

As the cf-DNA in a pregnant woman is a mixture of genomic DNA fragments of maternal and placental origin (cff-DNA), the fetal fraction represents a critical and limiting factor that seems to be reliably measurable from the tenth week for clinical purposes, when it reaches a proportion of about 10% progressively increasing in the following weeks.

Given its high sensitivity and specificity, the cost-effectiveness of and the absence of risk of pregnancy loss associated with amniocentesis or chorionic villus sampling, NIPT has turned out to be a widespread screening tool for the most common fetal aneuploidy. NIPT, in fact, results in being especially effective in detecting trisomy 21 (Down’s syndrome; 99% and 99.92%, respectively), trisomy 18 (Edwards syndrome; 96.8% and 99.85%, respectively), trisomy 13 (Patau syndrome 92.1% and 99.8%, respectively) and sex chromosome aneuploidies (with a lower sensitivity than the previously reported autosomal aneuploidies), with a combined false-positive rate (FPR) of 0.13% [3]. However, NIPT cannot be considered as a “prenatal diagnostic test” but, up to now, a screening tool, albeit a highly valuable one. In fact, the incidence of false-positive results, generally attributable to mosaicisms confined to placental (CPM) or, less frequently, to other conditions such as vanished twin, maternal CNVs, apoptosis of maternal cancer cells and fetal mosaicism, requires an invasive test (preferably, through an amniocentesis) to confirm a positive NIPT result [4]. Such circumstances may occur and, to date, represent the origin of possible discordant test results, failures and contraindications. However, it is expected that technological advances and the involvement of fetal medicine specialists and genetic counsellors, especially in the most complex cases, should allow current limitations to be overcome in the near future.

In the current state of our knowledge and available technical tools, the fact that the cff-DNA concentration is directly proportional to gestational age and inversely proportional to maternal body mass index (BMI) [4] represents a limitation to be kept in mind, and a reason to proceed with caution when proposing this screening method at too early a gestational age (i.e., in the first trimester) or to overweight women, potential causes for a low cff-DNA fraction and, consequently, no-call results or false negative. In that regard, studies accounting for more than 16,000 pregnancies found a low fetal fraction in maternal bloodstream to be linked to a higher risk of fetal aneuploidies [5,6].

The possibility of not having results or to obtain ambiguous results to be confirmed by other methods should be adequately clarified before applying the test, possibly in a prenatal genetic counseling setting to enable informed choices. 

In the current practice, the clinical application of NIPT is limited to the screening of the most common fetal aneuploidy and the associated risk assessment. However, in light of the considerable technological advancements in the field and progressively greater affordability of such techniques, NIPT has also begun to be used to test for single-gene disorders [7], as well as for additional and more rare chromosomal disorders [8] and sub-microscopic CNVs [9]. Although further research and validation are needed in order to determine the accuracy in detection rates and false positive and negative rates for these technical and clinical implementations, it is reasonable to predict that in the next few years NIPT will gradually become more applicable and widely adopted to the screening of several more genetic anomalies, including CNVs and single-gene disorders [10]. 

The suitability of advanced technical tools will involve further changes in clinical practice and, at the same time, will require an equally rapid ability to reflect on the ethical and legal aspects they open up.

## 2. Non-Invasive Prenatal Testing (NIPT) Ethical, Legal and Social Implications: Maybe Advancing Reproductive Autonomy, But at What Cost?

First introduced in 2011, NIPT was at first offered by private health care providers, and has in recent years been integrated among public healthcare services. In Italy, the NIPT test was introduced in Tuscany in 2019 as a public healthcare tool in the birth path [11]. 

The increased use of NIPT has significantly reduced the number of invasive tests carried out in many of the nations where it is commonly used. As of today, NIPT is used in less than 25% of pregnancies in most European countries, and as low as 5% in some countries. The highest levels of NIPT use in European Countries have been recorded in Belgium (>75% of pregnancies) and the Netherlands, Italy, Spain and Austria (generally ranging between 25% and 50% of pregnancies) [12]. In the USA, NIPT for high-risk patients (i.e., >35 years, positive cFTS, etc.) is covered by most healthcare insurance companies and most state-sponsored Medicaid programs (in all but nine states). Overall, at the national level, 25–50% of all pregnant women undergo NIPT, which mostly screens for chromosome 13, 18, 21 and sex chromosome aneuploidies. On the other hand, the ever lower costs of new molecular technologies and the non-invasiveness of the diagnostic method could allow for greater diffusion and availability, particularly in low-income developing countries.

Screening for other triploidies, rare aneuploidies and some microdeletions is also available, albeit rather rarely used, and not recommended.

Nonetheless, in terms of ethical, legal and social implications, NIPT has a broad range of complexities that need exploring. Prenatal screening for fetal abnormalities is a morally sensitive practice, since it could lead, or at least contribute, to a decision to terminate one’s pregnancy in case of positive results.

Although population screening programs are mostly directed towards the purpose of reducing morbidity and mortality rates, linked to diseases or disorders, in the population at large, two major ethical issues arise as a result of that objective, as far as prenatal screening is concerned. First and foremost, if the success of the program is deemed to rest upon the termination rate of fetuses with detected abnormalities, confirmed by follow-up testing, that interpretation might well generate a certain degree of pressure on women to seek to terminate their pregnancies if fetal genetic conditions are detected. Some fear the risk of turning the personal choice to voluntarily terminate one’s pregnancy into a “public health instrument” [13]. Secondly, the main objective of prenatal screening techniques makes the practice disputable according to the “disability rights” or “expressivist” critique. Such a philosophical stance denounces that a discriminatory message may be sent by the mainstream use of prenatal screening about the inherent worth of human life, and particularly the lives of those suffering from severe disabling conditions [14]. The considerable discrepancies that are still unsolved between the various assessments of NIPT’s potential benefits and risks, in addition to the ethical values to which they relate, are bound to engender challenges and conflicts that public policy-makers need to address. 

Three core principles have been laid out in a recent, wide-ranging analysis by the Nuffield Council on Bioethics [15], which may be summarized as follows:

- The societal context and environment in which NIPT screening is developed and provided ought to be taken into account when devising and undertaking NIPT policy-making initiatives.
- Access to NIPT should be guaranteed to women and couples under conditions and in an environment that prioritizes autonomous, informed choices.
- Potential, significant risks posed by the growing use and development of NIPT ought to be dealt with and minimized through concerted, multilayered efforts, involving all stakeholders and elected officials.

Such major ethical concerns have also been addressed and countered in policy positions issued by various societies in several countries (such as France [16], the United Kingdom [17], Denmark [18], and the Netherlands [19]). In their analyses, said institutions stressed that the fundamental purpose of prenatal screening is not to prevent children with specific abnormalities from being born, but rather to foster reproductive awareness and uphold reproductive rights by enabling pregnant women and their partners to exercise their full autonomy through informed decisions. 

## 3. More Information Fosters Procreative Autonomy? Not So Fast...

Not surprisingly, one of the most pressing ethical concerns related to NIPT is the opportunity to rely on informed decision making. Professional guidelines currently recommend that patients who opted for prenatal screening should receive pre- and post-test genetic counseling [20,21], as it is already the case at most academic medical centers worldwide and mandatory in the Italian national health system. In the case of NIPT, moreover, the risk of “routinization” or “trivialization” appears even greater than in other prenatal diagnostic approaches. That is likely due to the lack of risk for pregnancy by a non-invasive approach and the great simplicity of the clinical approach. Just by virtue of the higher degree of safety of this approach when compared to other prenatal tests, the risk is that all the important implications that NIPT entails may not be fully understood and weighed, if not thoroughly discussed in an informed decision-making process prior to the procedure.

If women are not offered clear information to help them make up their minds about NIPT or other prenatal screening techniques, about the risks and benefits of different approaches and the implications of all possible outcomes, they may not be able to think adequately and determine whether they really want the test results or how they would react to them. Data from experiences with a “standard” blood draw such as serum screening seem to point to poor levels of informed consent before the screening, with many patients remarking that they did not really want to be tested or that, on the contrary, they refused to without being fully aware of its function [22].

Furthermore, wide-ranging emotional and social implications could arise from unexpected test results, even to the point of negatively affecting reproductive autonomy. If the current Italian and, in particular, in Tuscany, two-step prenatal screening approach (a “combined test” followed by invasive test in case of high risk of aneuploidy) were entirely supplanted by NIPT, such a development would arguably detract from the twofold opportunity to foster informed decision-making. It may be argued that NIPT would likely lower health care costs, if it were to become a primary screen; nonetheless, it would also reduce the consultations and dialogue to only one point of contact between a pregnant woman and her physician to discuss the implications of undergoing prenatal testing [23]. 

Moreover, a large and growing availability of information (as the techniques and the scope of NIPT are further developed) could predict not only late onset diseases (such as cancers or degenerative diseases), but also predispositions to severe and common conditions (cancer, diabetes etc...). In addition to that, new methods based on non-coding RNA have been substantially developed [24]: research has focused on RNA-based non-invasive biomarkers, including cell-free nucleic acids such as miRNA, lncRNA and circRNAs [25,26], so as to detect some of the most common conditions related to gestation, such as congenital heart diseases, gestational diabetes and preeclampsia, among others. 

Moreover, associations have been widely researched between circulating small and long non-coding RNAs (ncRNAs) and cancer [27,28]. In this way, the so called incidental findings (i.e., off-target results with potential medical implications) can arise with more probability both for the fetus (minor anomalies or irrelevant information from a medical standpoint, such as paternity) and the mother (unsuspected maternal malignancy in asymptomatic women).

Certainly, such a wide-ranging enhancement of testing capabilities might be welcomed as beneficial in terms of upholding the reproductive autonomy of parents. However, there are significant concerns stemming from such unprecedented access to information about a human being yet to be born. Let us consider the confusion likely to be generated by such a deluge of genetic information, often hard to fully interpret and explain for physicians and for parents to fully understand [29]. Secondly, it is worth bearing in mind that even when NIPT does not lead to voluntary termination of pregnancy, there are serious privacy concerns for the future child as well as the mother, stemming from such a broad range of extensive genetic information. Some analysts have contended that testing purely for information on a range of conditions (e.g., adult-onset diseases) is morally objectionable, in that it could harm the child’s and parents’ ability to make future choices in an unconditioned fashion (the “right to an open future”, albeit not deemed an absolute right [30]). That could, to some extent at least, do damage, by exposing the child to higher levels of anxiety as a result of his or her awareness of a future with a particular condition [31]. 

## 4. Could NIPT Mainstreaming Go in the Direction of Making Selective Abortion Ethically Acceptable? The “Slippery Slope” Risk

Many have argued that larger and larger amounts of genetic information might ultimately lead to higher voluntary abortion rates [32] (although there seems to be no conclusive evidence to support that assumption [33,34]), as well as its discounting or ‘trivialization’, which could result in the choice to terminate a pregnancy on relatively flimsy grounds [35]. There is no denying that the earlier women have access to diagnostic information, the earlier a choice can be made, and the termination can take place in a way that is safer and relatively less emotionally traumatic [36]. After all, unlike invasive forms of testing such as amniocentesis, about which the decision not to test could be rationally warranted by the intention to avoid the higher risk of miscarriage, NIPT can potentially and significantly alter the decision-making process by increasing the pressure to test. Women would, therefore, be left with almost no reasonable motivation for refusing to undergo testing, other than their unwillingness to do so. However, that could entail a risk: should the NIPT unveil a high risk of Huntington’s disease, a woman may choose to terminate her pregnancy in the first trimester (a choice she may not have made in the second trimester?) based on the test’s result. NIPT has not yet been validated as diagnostic tool. In addition, testing for non-medical traits (e.g., the sex of the fetus) at an early stage could lead to abortions being used as a “selection tool”, which is extremely controversial from an ethical perspective. Some believe that the large-scale, long-term application of NIPT techniques and their possible further development and validation as a diagnostic test (thus avoiding the potential complications and invasiveness of chorionic villus sampling or amniocentesis) could likely produce a growth in both the rates of detection of important fetal conditions and in termination rates. Such a dynamic would in turn likely lead to a considerable reduction in the incidence of certain genetic conditions overall [37], and to fewer losses of normal pregnancies [38]. Quite possibly, such a development may be viewed by some as beneficial from the standpoint of public health, particularly in terms of bringing down the health care costs associated with disabilities [39]; still, studies have denounced the risk of creating even worse stigmatization and discrimination against those who live with such syndromes through no fault of their own [40]. In fact, some fear that the broad availability of NIPT would give rise to high social pressure on women who choose to give birth to a child suffering from genetic syndromes detectable by NIPT. That would be due to the fact that such burdensome conditions could be increasingly perceived as “easy to avoid” through termination of pregnancy [41]. Families of people with Down’s syndrome have expressed their worries [42] about a lower degree of social inclusion and understanding of Down’s syndrome sufferers [43], which might even lead to lower social and financial support for such families [44,45]. That development could even adversely affect research into the development of treatments for such conditions.

Making testing available indiscriminately, and dramatically broadening its scope and applications, could trivialize and discount the gravity of the decision to terminate a pregnancy, possibly resulting in different expectations and pressure about what women “should” decide, when they learn that their babies have genetic anomalies, or even traits deemed ‘undesirable’ by society. Hence, in light of the aforementioned dynamics, could NIPT, or other future simple and fast screening procedures for detecting fetal conditions, lay the groundwork for a society in which the “quest for a perfect baby” becomes acceptable and even mainstream? There are conflicting views on that point: some bioethicists have called attention to the risk of potentially creating new, biotechnology-based forms of eugenics, while others have cited the “Principle of Procreative Beneficence” (PB), according to which couples who choose to have children hold a moral reason, even an obligation, to select the child who, by virtue his or her genetic qualities and endowment, can have the best chances of achieving self-fulfillment in life [46]. Such a position represents a key element of transhumanism, but has been criticized by many who have seen in this “obligation” to create the best possible children, the morally reprehensible willingness to eliminate the disabled [47]. Hence, the selection for non-disease genes should be encouraged even if it may contribute to increasing social inequality [48]. With NIPT poised to achieve fetal whole-genome sequencing (WGS), i.e., a growing amount of genetic information obtained from cff-DNA, such concerns are unlikely to be allayed any time soon [49].

## 5. High Medicolegal Standards Must Be Met in Order to Avoid Negligence and Malpractice Allegations

Most litigation instances involving prenatal testing arise from negligence allegations, i.e., the breach of legal duty that resulted in “unreasonable risk” [50], which is common in virtually all medical malpractice-related litigation. Healthcare operators are typically charged with failing to provide services that meet the standards of reasonable professional practice governing the healthcare provider’s profession or specialty in force when the intervention was rendered. Plaintiff patients often allege that as a direct result of such non-compliance, damage was caused that would not otherwise have happened. 

Lawsuits centered around allegations of malpractice in the provision of prenatal genetic testing may arise under several circumstances. Physicians or genetic counselors may be accused of negligence in the provision of genetic counseling, e.g. withholding information from patients as to the potential reproductive risk based on carrier status or age, denying requests to carry out invasive procedures or neglect informing the patient about the need or availability of such procedures. As an integral part of the informed consent process, doctors are professionally bound to disclose and discuss risks, benefits, and possible alternatives to a procedure and may, therefore, have a duty to disclose the availability of NIPT. Explaining and discussing possible alternatives constitutes a fundamental element of the disclosure process; patients, in fact, are often in no position to assess the risk in abstract terms and, therefore, need to be provided with a frame of comparison, so that they can arrive at an informed decision [51]. Non-invasive screening options ought to be disclosed to patients prior to the implementation of an invasive procedure, especially when the non-invasive tests could give information that could help patients decide whether or not to consent to the procedure [52]. Patterns may also develop involving laboratory negligence charges or genetic counseling based on misinterpreted laboratory results which lead the patients to make choices that they would not have made, had they had correct information, including the choice to bring the pregnancy to term. These are usually referred to as “wrongful birth” or “wrongful life” instances. Laboratory negligence or genetic counseling based on incomplete or misinterpreted laboratory results may also lead to claims in which the plaintiffs argue that they would not have aborted a fetus had they been correctly diagnosed or properly counseled about the risks of terminating a pregnancy without further confirmatory test results. Such cases have been termed “wrongful abortion” lawsuits. Wrongful birth claims are ethically contentious: if in fact a pregnancy is terminated because of predicted anomalies, a conflict may come into being between the reproductive rights of women and the respect for the disabled [53]. Such conflict is further amplified if physicians can be held legally accountable for failing to recommend prenatal screening to their patients, and if a child is eventually born with a genetic condition that could have been foreseen. For that reason, prenatal screening and lawsuits centered around the notion of wrongful birth are criticized by several ethicists [54] and advocates for the rights of disabled people [55]; wrongful birth claims, they contend, demean or ignore the multiple unique traits and values of personhood that every newborn child brings into the world [56], including children suffering from genetic abnormalities resulting in disabilities. 

## 6. Conclusions: A Well-Balanced Approach Is Essential and Just as Hard to Define

In order to ensure an ethically sound implementation of NIPT, and likely non-invasive prenatal diagnosis (NIPD) in the near future, and to avoid a dangerous downslide towards selective reproductive technologies (SRTs) and eugenics, it is highly advisable that such issues be discussed by involving all stakeholders and the public at large, in a multidisciplinary coordinated approach. Foreseeably, the number of women availing themselves of NIPT screening will progressively rise, as will the number of conditions being tested. That undoubtedly represents a great opportunity for prospective mothers to make informed reproductive decisions, but it also poses issues and concerns about the routinization of testing and negative impacts on informed consent and decision making at its core. In order to ensure that each step towards greater control over human reproduction is suitably confronted, and to stave off any potential conflict with bioethical and social values, specific issues have to be addressed keeping an eye on the diversity in cultural contexts. After all, medically-assisted reproduction (MAP) techniques, which also entail legal and ethical challenges, have followed similar dynamics as they have developed over the years. Eventually, legislative frameworks have been enacted, at the national level, to govern MAP, with varying degrees of restriction [57]. With that in mind, newly updated frameworks should be developed, aimed at serving as guidance towards an ethically sustainable implementation of NIPT. Moreover, NIPT counseling must be provided in a way that prioritizes and upholds the rights and best interests of fetuses, pregnant women and couples, while at the same time avoiding paternalistic or directive initiatives by counselors, in keeping with the best traditional approaches of genetic counseling. In that regard, healthcare professional training, education and practice are of critical importance. 

## Data Availability

The datasets used or analyzed in the present study are available upon demand from the corresponding author.
